# From Operating Room to Intensive Care: A Case of Enterococcus Endocarditis

**DOI:** 10.7759/cureus.70154

**Published:** 2024-09-25

**Authors:** Nava R Sharma, Madalasa Pokhrel, Prakriti Lamichhane, Margaret Kuhn-Basti

**Affiliations:** 1 Internal Medicine, Maimonides Medical Center, Brooklyn, USA; 2 Medicine, Manipal College of Medical Sciences, Pokhara, NPL; 3 Infectious Disease, Maimonides Medical Center, Brooklyn, USA; 4 Pathology, Kathmandu Institute of Science and Technology Medical College, Lalitpur, NPL

**Keywords:** acute bacterial endocarditis, aortic valve insufficiency, infective endocarditis, surgery in infective endocarditis, treatment failure enterococcus endocarditis

## Abstract

Infective endocarditis is a serious infection of the endocardial surface of the heart, most commonly involving the heart valves. *Enterococcus* species, particularly *Enterococcus faecalis*, are significant but less common causes of infective endocarditis. *Enterococcus* can become pathogenic, especially in immunocompromised patients or those with recent surgical interventions. Due to the bacteria’s intrinsic resistance to many antibiotics, including cephalosporins, and low-level resistance to aminoglycosides, the treatment can be complicated. This case illustrates the progression of a patient from a relatively routine gynecological procedure to severe complications, including infective endocarditis, severe aortic regurgitation, and a cerebellar stroke. It also highlights the importance of vigilant monitoring for embolic events, particularly in those with recent surgical history, and the critical role of a multidisciplinary approach in managing such complex clinical scenarios.

## Introduction

Infective endocarditis is a serious and potentially life-threatening condition characterized by the infection of the endocardial surface of the heart, most commonly involving the heart valves [1]. *Enterococcus faecalis*, a gram-positive bacterium that is part of the normal gastrointestinal tract flora, has emerged as a notable pathogen in infective endocarditis, particularly in patients with underlying risk factors or recent surgical interventions [2]. Diagnosing *Enterococcus* endocarditis can be challenging initially due to its indolent presentation, but it can be later associated with a high rate of complications, including heart failure, systemic embolization, and stroke. The clinical course of infective endocarditis can be insidious, with initial symptoms often nonspecific, making early diagnosis challenging [3].

We present the case of a 43-year-old female who developed *Enterococcus faecalis* bacteremia following a robotic myomectomy performed a few weeks prior. Initially presenting with fatigue, abdominal pain, and chills, she was found to have splenic and small bowel infarcts, which were thought to be embolic complications from her recent surgery. Her condition subsequently worsened, leading to a diagnosis of infective endocarditis. This resulted in severe aortic regurgitation and culminated in a cerebellar stroke, marking a critical and life-threatening turn in her clinical course.

## Case presentation

A 43-year-old woman with a significant medical history experienced a complex and life-threatening series of events. Her medical history included obesity, which had been managed with a sleeve gastrectomy six years earlier, and chronic iron deficiency anemia treated with oral iron supplements. The patient had a three-year history of on-and-off heavy menstrual bleeding caused by uterine fibroids, for which she underwent a robotic-assisted myomectomy. The surgery was successful, with expectations of postoperative pain and a gradual return to her baseline health.

Eighteen days after the myomectomy, the patient presented to the emergency department (ED) with fatigue, abdominal pain, and chills that had persisted for three to four days. She denied experiencing headaches, changes in vision, chest pain, shortness of breath, nausea, vomiting, constipation, dysuria, or leg pain. Physical examination revealed tenderness in the left upper quadrant (LUQ) on deep palpation, but she was afebrile, and her vital signs were stable.

**Figure 1:**
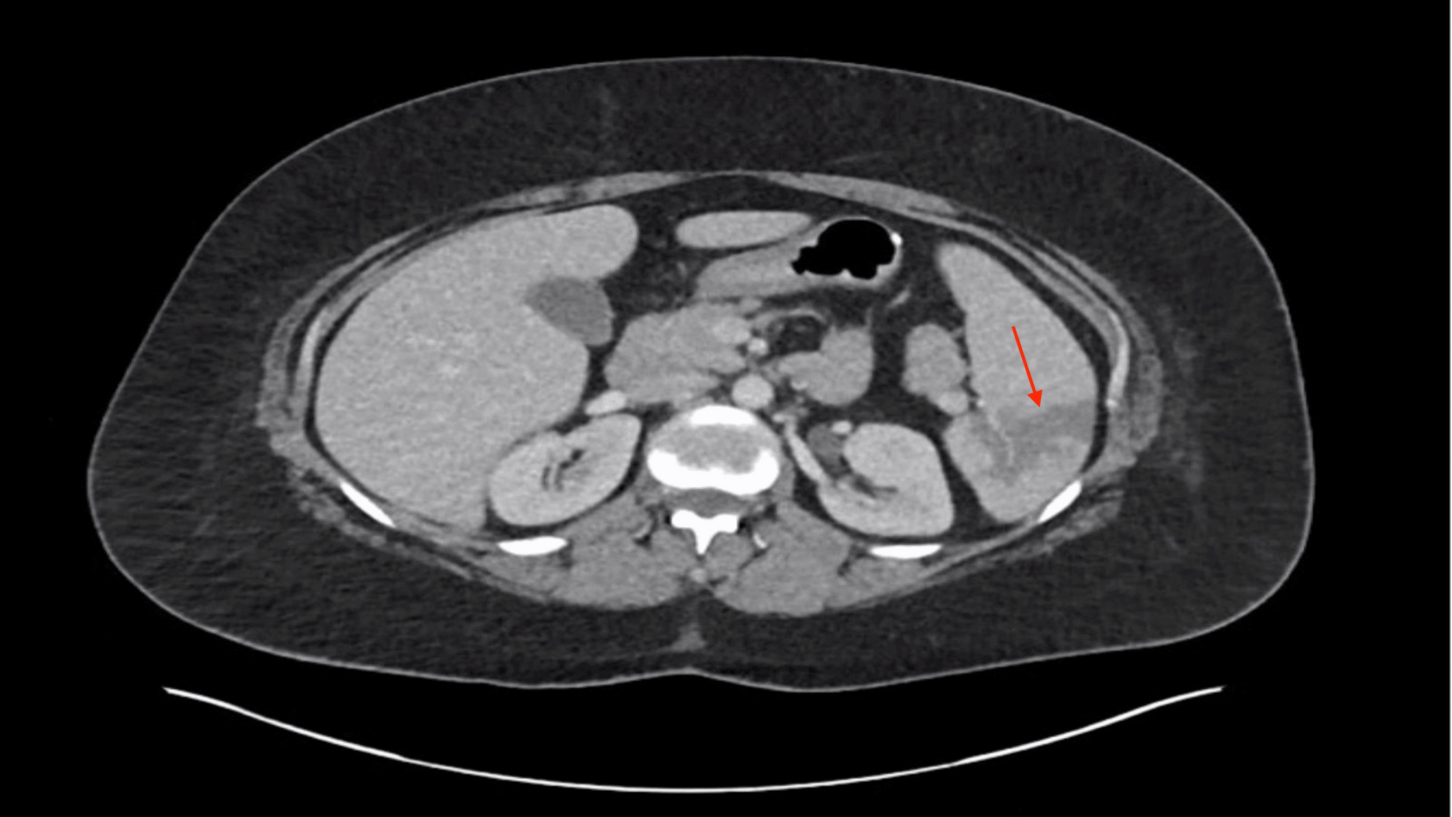
A CT scan of the abdomen showing a splenic infarct (red arrow).

Routine laboratory values were unremarkable, and the patient’s hemoglobin remained stable throughout the hospital stay. Iron studies and vitamin B12 and folic acid levels were normal. A CT scan of the abdomen and pelvis revealed a splenic infarct, small bowel ischemia, and edema with hypoenhancement in the small bowel, suggesting possible ischemia, as shown in Figure 1. Given her recent surgery, these findings were initially thought to be likely embolic infarcts. Blood cultures, echocardiography, and further workup for potential embolic sources were initiated. The patient was started on heparin for anticoagulation while the evaluation for cardiac and infectious sources continued.

The hematology team agreed that the infarcts were likely embolic and recommended continuing heparin with the possibility of transitioning to Eliquis if no surgical procedures were anticipated. They also advised ruling out a patent foramen ovale and infective endocarditis, with follow-up echocardiography and blood cultures. If no etiology was identified, they suggested a hypercoagulable workup, including tests for anticardiolipin antibodies, β2-glycoprotein, and lupus anticoagulant. A general surgery consultation recommended symptomatic management for the splenic infarct, with no immediate surgical intervention required.

The following day, the patient developed a fever while still receiving therapeutic heparin. Blood cultures returned positive for gram-positive cocci, later identified as *Enterococcus faecalis*. Initially, she was started on ampicillin, but due to concerns about bowel ischemia, her antibiotic regimen was changed to ampicillin/sulbactam 3 g IV every six hours to cover anaerobes. Despite these developments, imaging studies revealed no valvular pathology, and the patient remained hemodynamically stable, though she experienced mild LUQ pain during inspiration.

Five days after admission, the patient was discharged with a plan for outpatient follow-up. She was prescribed amoxicillin-clavulanic acid 875 mg orally every 12 hours for 10 days to complete a 14-day course of antibiotics for her bacteremia. Blood cultures were to be repeated to ensure clearance of the bacteremia, and she was scheduled for follow-up with her infectious disease (ID) and hematology teams.

**Figure 2:**
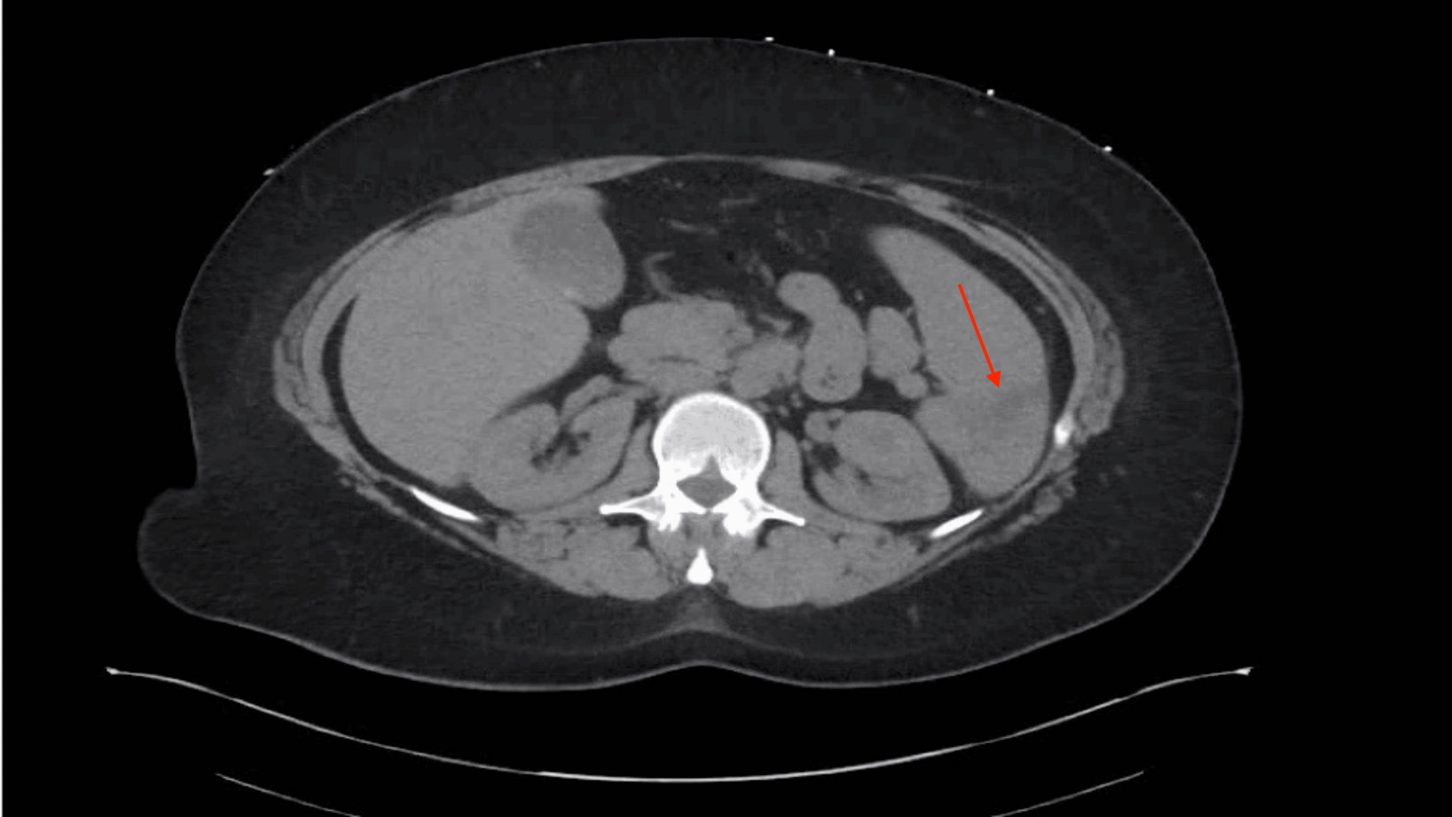
A repeat CT scan of the abdomen showing a resolving splenic infarct (red arrow).

Twenty days later, during her follow-up with the ID team, the patient had completed her antibiotic course and exhibited no systemic signs or symptoms of infection. Blood cultures were negative, confirming the clearance of the bacteremia. The following day, a hematology follow-up revealed a negative thrombophilia workup, including tests for prothrombin gene mutation, factor V Leiden mutation, and anticardiolipin antibodies, with normal homocysteine levels. The patient was advised to continue anticoagulation with oral apixaban. One week later, a follow-up visit with general surgery revealed that a repeat CT scan of the abdomen showed a resolving splenic infarct, as shown in Figure 2.

**Figure 3:**
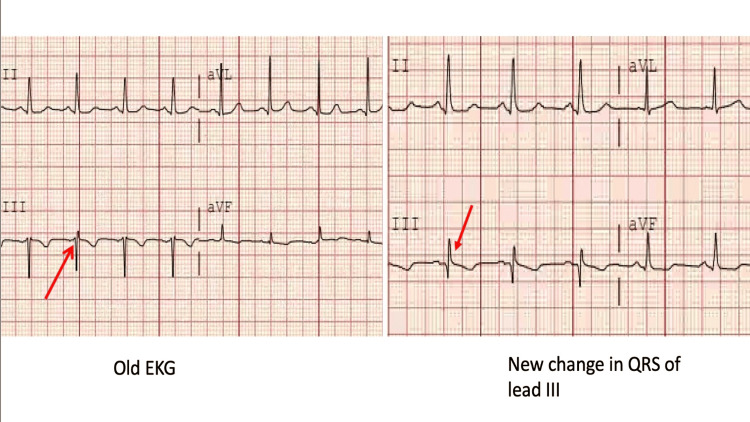
EKG showing new changes in lead III.

Approximately six weeks after her initial presentation, the patient returned to the ED with symptoms of dizziness, described as the sensation of the ground spinning beneath her feet, along with tingling in the fingers and toes of both her hands and feet. She also reported intermittent vertigo that had persisted for three weeks, associated with daily frontal headaches. Additionally, she described intermittent paresthesia in both unilateral and bilateral fingers and toes, with no clear provoking factors. She denied fevers, chills, shortness of breath, chest pain, weakness in her limbs, nausea, or vomiting. EKG also showed baseline changes in lead III compared to the EKG done four months back, as shown in Figure 3.

Given her symptoms, the differential diagnosis included benign paroxysmal positional vertigo, complex migraines, and acute ischemic stroke. A CT scan of her head revealed no acute hemorrhage or evidence of infarct, and CT angiography of the head and neck was unremarkable. However, an MRI of the brain revealed an acute/subacute infarct in the right cerebellum. A transthoracic echocardiogram (TTE) performed one day after her initial presentation showed a left ventricular ejection fraction of 66-70% and a mildly dilated left atrium. Given the new neurological findings, a repeat TTE with bubbles was ordered, which revealed new severe aortic regurgitation and a 0.7 cm mobile mass on the left ventricular surface, suggestive of aortic valve endocarditis.

**Figure 4:**
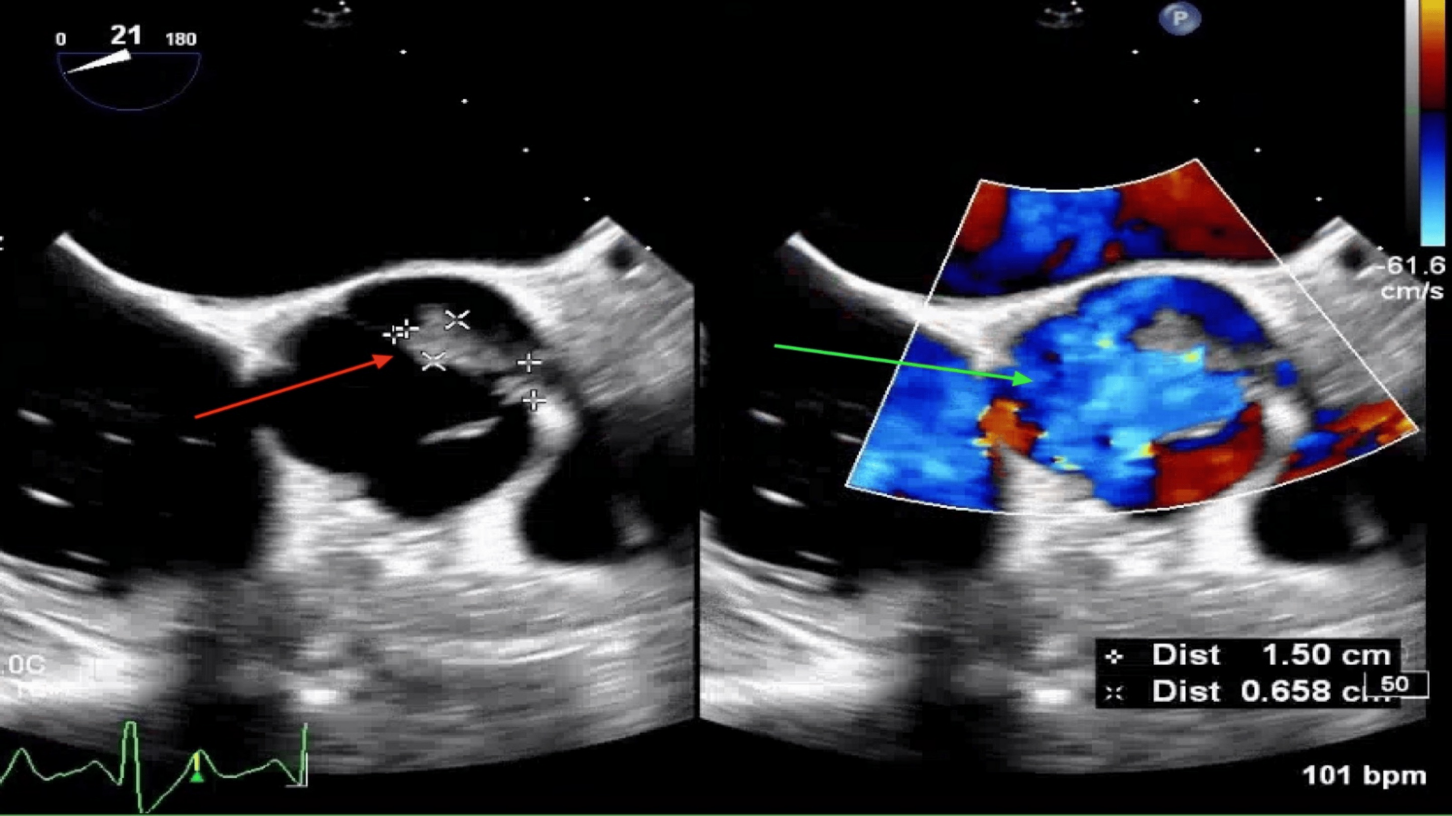
Transesophageal echocardiogram revealing a 15 × 6 mm vegetation at the tip of the left coronary cusp of the aortic valve (red arrow) and severe aortic insufficiency (green arrow).

A subsequent transesophageal echocardiogram revealed a 15 × 6 mm vegetation at the tip of the left coronary cusp of the aortic valve, causing severe aortic insufficiency with holodiastolic flow reversal in the descending aorta, as shown in Figure 4. No vegetations were observed on other valves, and the left ventricular function was normal. A bubble study was negative.

Blood cultures were again positive for *Enterococcus faecalis*, confirming the diagnosis of infective endocarditis. The patient was started on ampicillin 2 g IV every four hours and ceftriaxone 2 g IV every 12 hours. Given the severity of the aortic valve involvement, the patient underwent minimally invasive aortic valve replacement on the 16th day of admission. Postoperatively, she was discharged home on the 18th day of admission with a course of IV antibiotics to complete her treatment.

## Discussion

*Enterococcus* species, particularly *Enterococcus faecalis*, are significant but less common causes of infective endocarditis, accounting for approximately 10% of all infective endocarditis cases [3,4]. Despite being a part of the normal gastrointestinal and genitourinary flora, *Enterococcus* can become pathogenic, especially in immunocompromised patients or those with recent surgical interventions [3]. The bacteria’s ability to adhere to damaged endothelium, coupled with its intrinsic resistance to many antibiotics, makes it a formidable pathogen in endocarditis, often leading to severe complications if not promptly diagnosed and treated [5].

The pathogenesis of *Enterococcus* endocarditis typically involves bacteremia that originates from the translocation of the organism across mucosal barriers. This translocation is particularly common during gastrointestinal or urogenital procedures, where the integrity of mucosal barriers may be compromised. Patients with recent surgical interventions, indwelling catheters, or prior antibiotic use are at higher risk of developing *Enterococcus* endocarditis [3-5]. The bacteria’s adherence to damaged cardiac valves or endothelium initiates the formation of vegetations, which are aggregates of platelets, fibrin, microorganisms, and inflammatory cells [5]. These vegetations can embolize, leading to systemic complications, including stroke, as seen in the present case.

Diagnosing *Enterococcus* endocarditis can be challenging due to its often indolent presentation. Patients may present with nonspecific symptoms such as fatigue, weight loss, or low-grade fever, making early recognition difficult [6]. The Duke criteria, commonly used to diagnose infective endocarditis, may not always be fulfilled in the early stages, especially in the absence of a detectable cardiac murmur or typical valvular lesions on echocardiography [7]. Blood cultures are essential for diagnosis, but the slow-growing nature of *Enterococcus* can delay the identification of the pathogen, thereby postponing appropriate treatment [8].

*Enterococcus* endocarditis is particularly challenging to treat due to the bacteria’s intrinsic resistance to many antibiotics, including cephalosporins, and low-level resistance to aminoglycosides [9]. The standard treatment regimen often involves a combination of a β-lactam antibiotic, such as ampicillin, and an aminoglycoside, such as gentamicin, to achieve synergistic bactericidal activity. However, in cases where aminoglycosides cannot be used due to resistance or toxicity, alternative regimens include the combination of ampicillin and ceftriaxone [10]. The duration of antibiotic therapy is typically extended, often requiring six weeks or more, to effectively eradicate the infection.

*Enterococcus* endocarditis is associated with a high rate of complications, including heart failure, systemic embolization, and stroke. In particular, the involvement of the aortic valve, as seen in this case, often leads to severe aortic regurgitation, necessitating surgical intervention. The prognosis of *Enterococcus* endocarditis is generally poor, with a mortality rate ranging from 15% to 25%, largely due to the complexity of the infection and the potential for delayed diagnosis and treatment [11].

This case illustrates the progression of a patient from a relatively routine gynecological procedure to severe complications, including infective endocarditis, severe aortic regurgitation, and a cerebellar stroke. It highlights the importance of vigilant monitoring for embolic events and the critical role of a multidisciplinary approach in managing such complex and evolving clinical scenarios.

## Conclusions

*Enterococcus* species, particularly *Enterococcus faecalis*, are less common causes of infective endocarditis but may lead to serious complications. *Enterococcus* endocarditis may develop in patients with recent surgical histories, even when initial presentations are nonspecific and diagnostic findings are equivocal. A high index of suspicion is needed for infective endocarditis in patients presenting with embolic phenomena, especially when *Enterococcus faecalis* is isolated from blood cultures. This case illustrates the critical need for a multidisciplinary approach in managing such cases, involving close collaboration between ID specialists, cardiologists, and surgeons to optimize outcomes. It also emphasizes the necessity of early and aggressive treatment, including the potential need for surgical intervention, to prevent catastrophic complications such as severe aortic regurgitation and stroke.
